# Modulation of Human Peripheral Blood Mononuclear Cell Signaling by Medicinal Cannabinoids

**DOI:** 10.3389/fnmol.2017.00014

**Published:** 2017-01-24

**Authors:** Wesley K. Utomo, Marjan de Vries, Henri Braat, Marco J. Bruno, Kaushal Parikh, Mònica Comalada, Maikel P. Peppelenbosch, Harry van Goor, Gwenny M. Fuhler

**Affiliations:** ^1^Department of Gastroenterology and Hepatology, Erasmus MC, Erasmus University of RotterdamRotterdam, Netherlands; ^2^Department of Surgery, Radboud University Medical CenterNijmegen, Netherlands; ^3^Department of Cell Biology, University Medical Center GroningenGroningen, Netherlands; ^4^Institute for Research in BiomedicineBarcelona, Spain

**Keywords:** kinome profiling, mTOR-S6, monocyte, T cells, inflammatory signaling

## Abstract

Medical marijuana is increasingly prescribed as an analgesic for a growing number of indications, amongst which terminal cancer and multiple sclerosis. However, the mechanistic aspects and properties of cannabis remain remarkably poorly characterized. In this study we aimed to investigate the immune-cell modulatory properties of medical cannabis. Healthy volunteers were asked to ingest medical cannabis, and kinome profiling was used to generate comprehensive descriptions of the cannabis challenge on inflammatory signal transduction in the peripheral blood of these volunteers. Results were related to both short term and long term effects in patients experimentally treated with a medical marijuana preparation for suffering from abdominal pain as a result of chronic pancreatitis or other causes. The results reveal an immunosuppressive effect of cannabinoid preparations via deactivation of signaling through the pro-inflammatory p38 MAP kinase and mTOR pathways and a concomitant deactivation of the pro-mitogenic ERK pathway. However, long term cannabis exposure in two patients resulted in reversal of this effect. While these data provide a powerful mechanistic rationale for the clinical use of medical marijuana in inflammatory and oncological disease, caution may be advised with sustained use of such preparations.

## Introduction

Although cannabis, derived from the dried flower buds of the female plant of *Cannabis sativa L*., has been used in medicine for many centuries, the recent decade has seen an increased acceptance of medical marijuana preparations for pain relief in a variety of inflammatory and oncological conditions (Gerich et al., 2015). Cannabis has been used in clinical practice for its analgesic, nausea-reducing and hunger-inducing properties and has recently been approved for treatment of spasticity caused by multiple sclerosis (Wilson and Nicoll, 2002). Cannabis contains several components, but its main psychoactive ingredient is Δ9–tetrahydrocannabinol (THC) (Howlett, 1995). Two cannabinoid receptors have been identified to date; CB1 and CB2 (Howlett, 1995), in addition to which the orphan G-protein coupled receptor 55 was shown to be activated by cannabinoids (Henstridge, 2012). CB1 is expressed both in the periphery and in structures of the central nervous system, where it accounts for the main effects seen after cannabis use, including sedation, dizziness, confusion and somnolence (Barinaga, 2001). Although CB2 receptors are also found and functional in the central nervous system, CB2 is mainly considered a peripheral cannabinoid receptor (Van Sickle et al., 2005). It is mostly found on cells of the immune system and is therefore speculated to play a part in immunoregulatory responses, although its mechanistic aspects remain to be elucidated (Turcotte et al., 2016).

Studies involving the potential role of cannabinoid receptors in inflammatory disease have shown that activation of the cannabinoid system can offer protection in experimental colitis models by reducing inflammation (Massa et al., 2004; Klein, 2005). In cerulein-induced acute pancreatitis, administration of cannabinoid agonists reduced inflammation and alleviated pain in mice (Michalski et al., 2007). Follow up research demonstrated that the addition of cannabinoids leads to a reduced inflammatory and fibrotic profile in pancreatic stellate cells *in vitro* (Michalski et al., 2008). Despite these indications of an effect of THC on immunity, very little is known of the direct immune modulatory effect of THC on peripheral blood cell populations. *Ex vivo* THC treatment reduces expression of cell surface receptors CD14, CXCR4 and CCR5 on isolated monocytes, thereby limiting HIV infection rate of these cells, but how cannabinoid receptor engagement provokes these effects remains unexplored (Williams et al., 2014). Mouse studies show that THC also exerts an effect on adaptive immunity, through modulation of T cell differentiation and reducing their interferon-γ production, but again the underlying effects on T cell biology remain obscure and require urgent clarification (Steffens et al., 2005; Karmaus et al., 2013).

Further hampering understanding of the processes mediating THC effects is the fact that although both CB1 and CB2 are Gi-protein coupled receptors, *in vitro* studies in mast cells show that these receptors mediate diametrically opposed effects on downstream adenylate cyclase activity and cAMP levels and thus the net effect of medical cannabis on signal transduction *in vivo* remains unclear (Small-Howard et al., 2005). In neuronal cells, cannabinoids were shown to decrease adenylate cyclase activity and cAMP levels, and activate the PI3 kinase/Akt, p38MAP kinase and ERK signaling cassettes, thereby modulating neuron-specific ion channels (Chiurchiù et al., 2015). In peripheral immune cells, however, activation of PI3 kinase/Akt, p38MAP kinase and ERK are more associated with an inflammatory response (van den Brink et al., 2000), whereas there appears to be a consensus that the effects of medical cannabis are of an anti-inflammatory nature (Mallat et al., 2013), suggesting that cannabinoid signaling in non-neuronal cells may be markedly different. Thus defining the action of medical cannabis on *in vivo* signaling of human immune cells is now a pressing concern, and essential for the design of novel rational cannabinoid therapy.

The above-mentioned considerations prompted us to explore the effects of medical marijuana using kinome profiling (Fuhler et al., 2011; Hazen et al., 2011), revealing wide-spread signaling effects of cannabis on peripheral blood mononuclear cells.

## Materials and Methods

### Collection of Human Materials and Treatment With Medical Marijuana Preparation

Patients suffering from chronic abdominal pain as a result of chronic pancreatitis or postsurgical pain were recruited at the Radboud University Medical Centre, Nijmegen, the Netherlands (Supplementary Table S1). The pain was considered severe enough for medical treatment, despite endoscopic, surgical or medical interventions. This study was part of two phase 2 trials using identical randomized, double-blind, placebo-controlled, parallel designs (clinicalTrials.gov ID: NCT01551511 and NCT01562483). For study controls unpaid cannabis-naïve healthy volunteers were solicited from the medical school student and employee population. For *in vivo* assessment of the effect of THC, blood for measurements was collected in Li-heparin-containing Vacutainer tubes (BD Vacutainer Systems, Plymouth, U.K.) at 0 and 180 min relative to drinking 400 ml of a medicinal cannabis preparation (Bedocran; Veendam, The Netherlands), prepared by covered boiling of 1 g Bedrobinol in 1 L of water for 15 min. For studies in patients, Namisol^®^ tablets containing purified, natural and standardized THC content were administered orally as add-on medication. The treatment regimen consisted of 2 phases: a step-up phase and a stable phase. In the step-up phase, patients received 3 mg Namisol^®^ three times a day (TID) for the first 5 days. If this dose was considered tolerable, the dose was increased on day 6 to 5 mg TID, and if not, the patient was withdrawn. The same procedure was conducted on day 9–10, tolerability was evaluated again and if 5 mg TID dosage appeared tolerable for the patient, the dosage was further increased to 8 mg TID from day 11. When 8 mg appeared to induce unacceptable adverse events, the dosage was tapered to 5 mg TID. This dose was maintained up to day 50–52 and was considered the stable phase. Blood samples were acquired on day 0 predose, day 15 predose, day 50–52 at 4 timepoints (predose, 1, 3, and 5 h after intake). For the graphic representation of the study design see Supplementary Figure S4.

### Peripheral Blood Mononuclear Cell (PBMC) Isolation and Stimulation

Peripheral blood mononuclear cells were isolated using density gradient centrifugation (Ficoll-Paque, GE Healthcare). Cells were washed in PBS twice, and either used directly, or resuspended in RMPI culture medium, supplemented with 10% fetal bovine serum and penicillin/streptomycin for *in vitro* stimulations. *In vitro* stimulations were performed in 96 wells plates (Corning, Tewksbury, MA, USA), using 100 ng/mL lipopolysaccharide (LPS, Sigma Aldrich, St Louis, MO, USA), 1.2 × 10^5^ CD3/CD28 Dynabeads (Life Technologies, Carlsbad, CA, USA) and/or 2 ng/ml pure THC (Echo pharmaceuticals, Nijmegen, the Netherlands) for the indicated time points. Concentration THC used was based on serum levels measured in individuals 2 h after Namisol^®^ ingestion (de Vries et al., 2016).

### Kinome Profiling

Kinome profiling was performed as described in (Fuhler et al., 2011). PBMCs were lysed in ice-cold Pepchip cell lysis buffer (20 mM Tris-HCl, pH 7.5, 150 mM NaCl, 1 mM Na2EDTA, 1 mM EGTA, 1% Triton X-100, 2.5 mM sodium pyrophosphate, 1 mM MgCl2, 1 mM μ-glycerophosphate, 1 mM Na3VO4, 1 mM NaF, 1 μg/ml leupeptin, 1 μg/ml aprotinin, 1 mM PMSF). Samples were sonicated four times for 5 s on ice and centrifuged at 7000 × *g* for 10 min at 4°C. Protein content in the clear supernatant was determined with a bicinchoninic acid protein assay kit (Pierce, Rockford, IL, USA), using BSA as the standard, and the supernatant was stored at -80°C until peptide array analysis.

For peptide array analysis, we employed the Pepchip kinomics array, featuring 960 different human-only kinase substrate peptides in addition to 70 positive and negative controls, each spotted in triplicate. In short, the cell lysates were cleared by centrifugation and peptide array incubation mix was produced by adding 10 μl of activation mix (50% glycerol, 50 μM ATP, 0.05% v/v Brij-35, 0.25 mg/ml bovine serum albumin) and 2 μl [γ-^33^P] ATP (∼1000 kBq; Amersham AH9968). Next, the peptide array mix was added onto the chip, and the chip was kept at 37°C in a humidified stove for 90 min. Subsequently the peptide array was washed twice with Tris-buffered saline with Tween 20, twice in 2 M NaCl, and twice in demineralised H_2_O and then air-dried. The chips were exposed to a phosphor screen for 72 h, and the density of the spots was measured and analyzed with array software (ScanAnalyze).

### Statistical Analysis

The peptide array data analysis was done as described earlier (Parikh et al., 2014). ScanAlyze software was used. Spot density and individual background intensities were analyzed using grid tools and data from three individual experiments, each consisting of three technical replicates, were exported to an excel sheet for further analysis. Means of spot intensity (i.e., kinase activity toward a specific substrate) were first subjected to Markov state analysis (i.e., is the peptide significantly more phosphorylated than background) and the reactions in which this is the case are listed in Supplementary Table S2, which also include other numerographic and biochemical information on the data. These Markov scores were collapsed on signal categories that provide insight into the difference in signaling between the THC-stimulated and unstimulated conditions.

### Flow Cytometry

Cells were fixed using 2% paraformaldehyde/1% FCS/0.02% EDTA in PBS and stored at 4°C. Surface staining with CD3-Amcyan (1:25; BD Biosciences, Breda, Netherlands) was performed the next day. Next, the PBMCs were permeabilized using a permeabilisation buffer (0.5% saponine, 1% FCS, 0.02% EDTA in PBS) and subsequently stained intracellularly with V450 Mouse anti-phospho S6 (pS6) (1:20; BD Biosciences, Breda, the Netherlands). Data was analyzed using FlowJo software (vX.0.6, Treestar, Ashland, OR, USA).

### Quantitative Western Blot Analysis

Immunoblotting of PBMC lysates was performed as described, with some adjustments (Somasundaram et al., 2013). After centrifugation, PBMCs were lysed in 2x Laemmli buffer (1% Bromophenol blue, 1% dithiothreitol, 10% sodium dodecyl sulfate, 1M Tris-Cl pH6.8) and boiled. Proteins were separated by polyacrylamide gel electrophoresis, and gels were blotted on Immobilon-FL transfer membrane (Millipore, Billerica, MA, USA). Primary antibodies against phosphorylated forms of p38, extracellular signal-regulated kinase (ERK), Protein kinase B/AKT, NF-κB-p65 and p70S6 were from Cell Signaling Technology (Beverly, MA, USA). Anti-rabbit or anti-mouse IRDye-conjugated secondary antibodies were used according to manufacturer’s directions, and blots were scanned by Odyssey infrared imaging (LI-COR Biosciences, Lincoln, NE, USA). Equal loading was confirmed by reprobing blots with β-actin antibodies (Santa Cruz Biotechnology, Santa Cruz, CA, USA). Quantification was performed using Odyssey 3.0 software. To calculate the relative pS6 levels, densitometry values of phosphorylated proteins were divided by densitometry values of the β-actin signal in the same lanes.

## Results

### Kinome Profiling of Lipopolysaccharide-Stimulated Blood Demonstrates Inhibition of Pro-inflammatory Signaling Following Medical Cannabis Use

There is little insight into the action of medical cannabis on signal transduction in human lymphocytes. Hence we asked three healthy cannabis-naïve volunteers to drink a medicinal cannabis preparation. Peripheral blood was obtained before and 2 h after ingestion. In order to mimic the inflammatory state commonly aﬄicting patients who are prescribed medicinal cannabis and as kinome profiling is most useful to characterize effects in potentiated signaling networks (Irish et al., 2004), blood was stimulated *ex vivo* for 10 min with 100 ng/ml lipopolysaccharide, followed by isolation of peripheral blood mononuclear cells (PBMCs) which were immediately subjected to kinome profiling (Diks et al., 2004). Kinome profiling constitutes an unbiased approach to characterizing signal transduction and in our case involves measuring the kinase activity toward 976 different substrates. Kinome profiles obtained before and after medical cannabis use were contrasted (Supplementary Figure S1 shows untreated versus medicinal cannabis-treated peripheral blood scatter plot where each dot represents the normalized mean phosphorylation level for each of the 976 substrates arrayed). As expected, there was a great deal of similarity between the two phosphoproteomes (*r*^2^= 0.88). However, a distinct subset of substrates falls off the diagonal toward the after medicinal cannabis axis (kinase activity stimulated by medicinal intake) or to the before medicinal cannabis axis. In fact, we found that phosphorylation of 124 substrates on the 976 peptide array differs significantly between the two data sets. Thus our results form the first documentation that *in vivo* treatment with medical cannabis acutely influences signal transduction in the human PBMC compartment.

**FIGURE 1:**
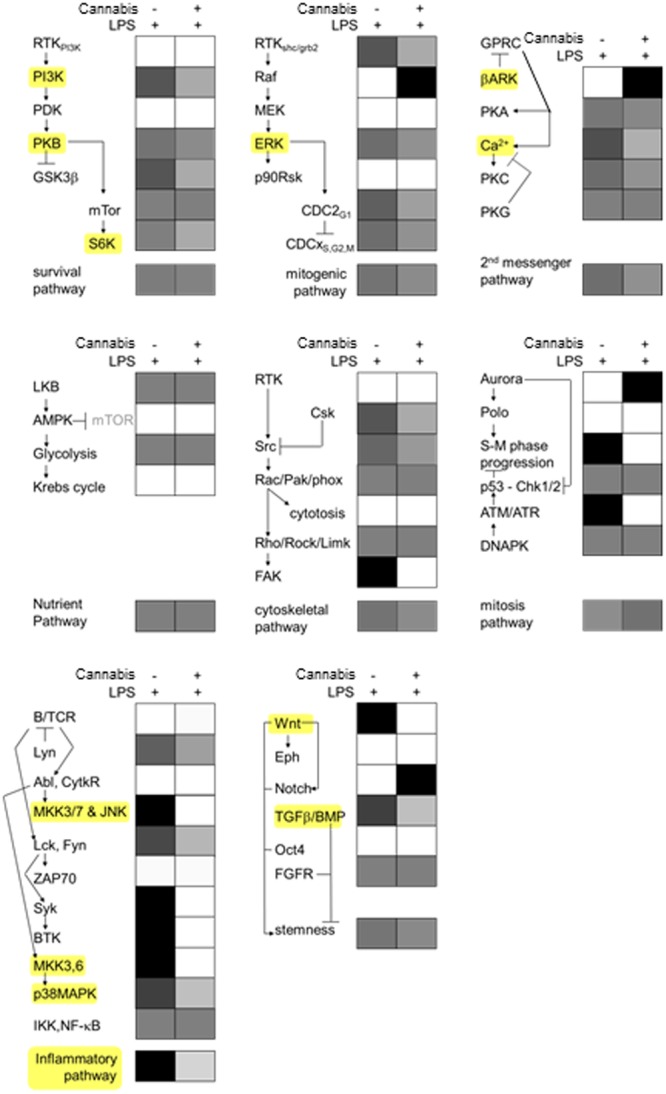
**Potentiated signal transduction in PBMCs before and after the intake of medical cannabis.** Blood was drawn from three self-reported cannabis-naive volunteers before and 2 h after the drinking of a medical cannabis preparation and stimulated for 10 min with lipopolysaccharide (100 ng/ml 10 min). Subsequently PBMC were isolated and investigated using peptide arrays exhibiting 976 different kinase substrates. Results were collapsed on elective signal transduction categories and expressed in gray scale (representing the fraction of peptides phosphorylated in either condition per pathway. White means no peptides were significantly phosphorylated and black means all phosphorylated substrates were found in one of the conditions). Some of the most prominently affected signal transduction elements have been highlighted in yellow. Note inhibition of pro-inflammatory signaling following medical cannabis use.

To obtain insight into the underlying effects, kinome results were collapsed on elective signal transduction categories and the potentiated kinome of cannabis unstimulated and stimulated PBMCs was contrasted. **Figure 1** shows the result and reveals a plethora of effects evoked by medical cannabis in the PBMC compartment. Generally speaking, G protein-coupled receptors evoke activity of receptor kinases that desensitize subsequent responses through β-arrestin-mediated mechanisms including phosphorylation, internalization, and receptor degradation (Sato et al., 2015). We observed strong induction of G protein-coupled receptor kinase activity (denominated as beta-adrenergic receptor kinase activity, **Figure 1**) following ingestion of medical cannabis by our volunteers. Other prominent effects include a downregulation of the pro-mitogenic kinase ERK and according signs of impaired cell cycling, which fits well with the anti-proliferative effects of cannabinoids on cancer cells *in vitro* (Chakravarti et al., 2014). The most significant effect, however, appeared to be a prominent downregulation of pro-inflammatory signaling, in particular that mediated by the stress-activated kinases p38MAP kinase and JNK, both prominent mediators of inflammatory gene transcription whose inhibition counteracts human inflammation *in vivo*, particularly for innate immunity (Hommes et al., 2002; Branger et al., 2003). In addition, deactivation of the PKB/mTOR signaling cascade and Ca^2+^ signaling was observed, whose inhibition is powerful in combating adaptive immune responses (e.g., in orthotopic organ transplantation recipients). The results would thus support the short-term use of medical marijuana in cancer (reduced cell proliferation) and autoimmune disease (diminished pro-inflammatory signaling).

### Direct Effects of THC on the PBMC Compartment

**FIGURE 2:**
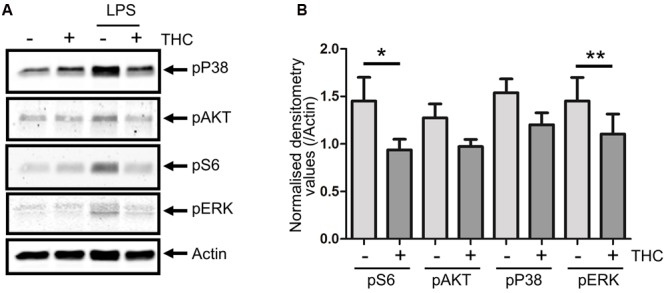
***In vitro* validation of direct THC effect on immune cells.** PBMCs were isolated from healthy subjects (*n* = 6) and after *in vitro* pretreatment with THC (2 ng/mL) for 1 h, cells were stimulated with 100 ng/mL lipopolysaccharide (LPS) for 10 min. Western blot analysis shows that unstimulated cells show relatively little activation of p38MAPK, AKT, S6 or ERK, while LPS induces phosphorylation of these kinases. Pretreatment of PBMCs with THC significantly reduces LPS-induced pro-inflammatory phosphorylation patterns. **(A)** Representative example is shown. **(B)** Quantification of phosphorylation levels of LPS-stimulated cells, normalized for total Actin levels are shown. pS6 and pERK levels are significantly reduced upon THC treatment of cells (^∗^0.0357, ^∗∗^0.0238). Phosphorylation of p38 was reduced upon THC treatment in 5/6 subjects. pAKT was detectable in only four subjects, in three of which phosphorylation was reduced in THC treated cells.

In our experimentation, drinking a medical cannabis preparation provoked acute strong anti-inflammatory signaling in volunteers. This set up of experimentation does not address, however, if these effects are mediated by THC, generally assumed to be the most potent bio-active compound in marijuana, or some other plant constituent. Furthermore, effects of the treatment can be the result of a direct effect on the PBMC compartment, but may also have been the result of an indirect effect mediated by secondary mediators, either neuronal or non-neuronal. Hence, we wished to validate the observed kinomic changes *in vitro* through another technique (**Figure 2**). Therefore, PBMCs were isolated from THC naive healthy subjects, pre-treated *in vitro* with 2ng/mL THC for 1 h and stimulated with lipopolysaccharide (LPS) for 10 min. As shown in **Figure 2A**, LPS effectively triggered phosphorylation of p38MAPK, ERK, S6 and AKT in PBMCs. Pretreatment of cells with THC significantly reduced these phosphorylation patterns (**Figure 2B**). Consistent with kinome profiling results, no effect of THC treatment was seen on p65 phosphorylation (indicating NFκB signaling; Supplementary Figure S2). LPS-mediated inflammatory signaling is directly reduced by interaction of THC with immune cells, probably through engaging CB1/2 receptors.

### Innate and Adaptive Immune Response Require Different Signals

**FIGURE 3:**
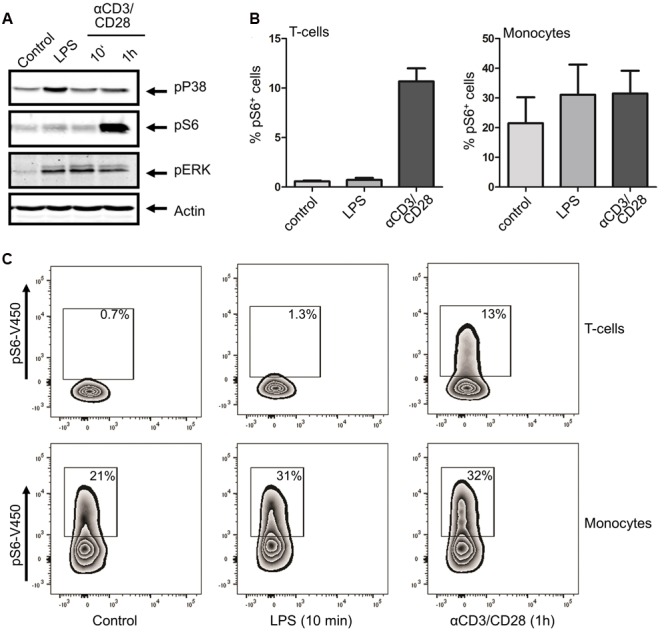
**Differential activation of immune signaling using monocyte or T cell –specific triggers.** Whole PBMC fractions were stimulated with either LPS (10 min) or αCD3/CD28 (10 min or 1h). **(A)** Western blot analysis shows that both stimuli induce a distinct set of phosphorylations. **(B)** Percentage of phospho-S6 positive cells as determined by intracellular FACS analysis, confirming that LPS does not trigger S6 phosphorylation in CD3^+^ T cells, whereas αCD3/CD28 generates a robust response in this cell type. In contrast, lipopolysaccharide does stimulate S6 phosphorylation in monocytes, with αCD3/CD28 also eliciting a response. **(C)** Representative example of FACS analysis of 4 independent experiments is shown.

LPS is generally regarded as an agonist of innate immune cells, most notably monocytes, with relatively little effect on adaptive cells (Janssens and Beyaert, 2003). However, in light of the potential effect of THC on T cells, we wanted to specifically investigate the effect of THC on inflammatory T cell signaling as well. To this end, we first confirmed innate and adaptive–specific signaling in PBMCs through stimulation with LPS and the T cell stimulus αCD3/CD28, respectively. Indeed, LPS drastically enhanced p38 phosphorylation in PBMCs, with little p38 activation seen upon αCD3/CD28 stimulation (**Figure 3A**). In contrast, while S6 phosphorylation was observed upon LPS stimulation, αCD3/CD28 was a much more efficient trigger of S6 phosphorylation. Considering the fact that PBMCs are a mixture of cells, the main constituents of which are monocytes and T cells, we confirmed these findings by intracellular flow cytometry of phospho-S6, allowing separate analysis of these cell subsets (see Supplementary Figure S3 for gating strategy). **Figures 3B,C** show that while LPS had little effect on S6 phosphorylation in CD3^+^ T cells, a robust stimulation of pS6 was observed in response to αCD3/CD28 stimulation. As constitutive S6 phosphorylation in monocytes was consistently higher than in T cells, LPS-enhanced S6 phosphorylation in this subset was more modest. Interestingly, αCD3/CD28 stimulation also resulted in S6 phosphorylation in monocytes, suggesting that T cell products released upon αCD3/CD28 stimulation in the PBMC mixture are capable of activating monocytes. These data suggest that while the innate pro-inflammatory response requires p38 activity, both the innate and adaptive immune responses are dependent on mTOR-p70S6-S6 signaling.

### THC Decreases mTOR-S6 Signaling Within CD3^+^ T Cells and Monocytes *In vitro*

**FIGURE 4:**
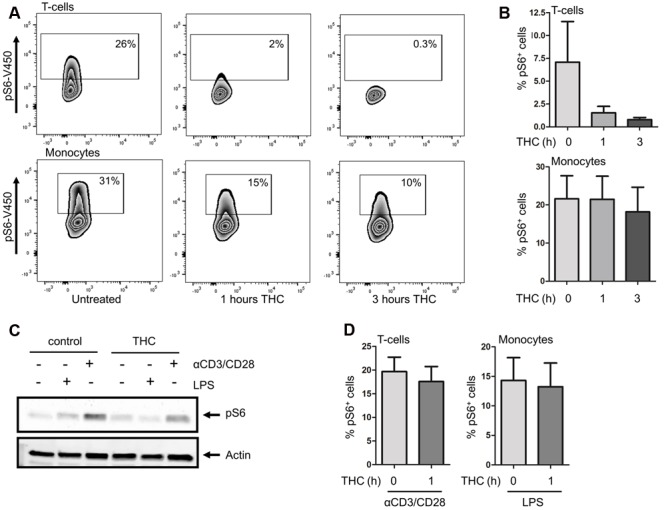
**THC reduces S6 phosphorylation in T cells and monocytes. (A)** Basal levels of S6 phosphorylation were determined by intracellular flow cytometry. S6 phosphorylation was reduced in monocytes and T cells upon *in vitro* treatment of cells with THC for 1 or 3 h. **(B)** Mean percentage of pS6 positive CD3^+^T cells (upper panel) and monocytes (lower panel) of six independent experiments. **(C)** Whole PBMC fractions were pretreated *in vitro* with THC for 1h or left untreated, and subsequently stimulated with either LPS (10 min) or αCD3/CD28 (1 h). THC pretreated samples show less S6 phosphorylation upon LPS or αCD3/CD28 stimulation (representative example of 6 experiments is shown). **(D)**. FACS analysis of LPS (10 min) or αCD3/CD28 (1 h) stimulated PBMCs, gated on monocyte and T cell fractions, respectively. Mean of four independent experiments is shown.

Our kinome data documents that the *in vivo* use of medicinal cannabis downregulates potentiated mTOR pathway activation in PBMCs, but leaves the cell types contributing to these effects unresolved. We thus decided to further investigate the effect of pure THC on the mTOR pathway in the different immune cell subsets constituting the PBMC compartment. To this end, isolated PBMCs of healthy volunteers were incubated for 0, 1, or 3 h with 2 ng/ml pure THC. As shown in **Figures 4A,B**, THC drastically reduces S6 phosphorylation in CD3^+^ T cells. Again, constitutive phosphorylation of S6 is higher in monocytes, and THC treatment results in attenuated levels of pS6 in the latter subset as well, although effects on the T cell compartment are much more marked. Next, we investigated the effect of THC on LPS- or αCD3/CD28-mediated S6 phosphorylation. **Figure 4C** shows that THC reduces both LPS- and αCD3/CD28-mediated pS6 in whole PBMC fractions as determined by Western blot analysis, which was qualitatively confirmed by separate analysis of T cell and monocyte populations by intracellular FACS analysis (**Figure 4D**). Thus medical cannabis acutely impairs signaling mediating both innate and adaptive immunity and thus our results would support the use of cannabis for treating patients suffering from an exaggerated immune response.

### Short Term Downregulation, But Potential Long Term Upregulation of the mTOR Pathway by THC in Patients *In vivo*

**FIGURE 5:**
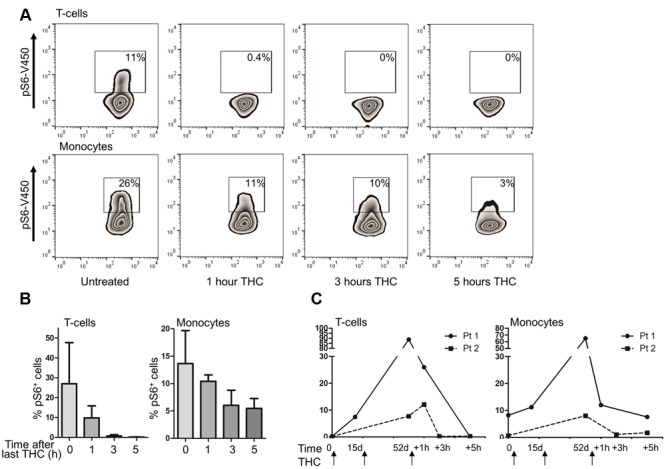
**Intake of THC reduces S6 phosphorylation in patient PBMCs in the short term, but enhances S6 phosphorylation in the long term. (A)** PBMCs were isolated from patients taking medicinal marijuana for pain-related complaints for 52 days. On day 52, samples were obtained pre-dose, and 1, 3, and 5 h after THC intake. A representative example of *n* = 4 is shown, demonstrating that the short term effects of THC intake include a decrease in S6 phosphorylation. **(B)** Mean percentage pS6 positive T cells and monocytes (*n* = 4). **(C)** For two patients, PBMCs were isolated pre-dosing on day 0, day 15 and day 52, as well as post-dose on day 52. Both patients show a clear increase of S6 phosphorylation during prolonged THC treatment, which is decreased upon short term treatment.

Having seen a strong immunosuppressive signaling in T cells and monocytes *in vivo* and *in vitro* in healthy subjects, we wondered whether these findings would also be observed in patients undergoing prolonged use of medicinal cannabis in the course of their treatment. We therefore measured S6 activity by intracellular FACS analysis in patients experimentally receiving oral natural THC as a tablet (Namisol^®^). Four patients were included in the study for severe abdominal pain, resulting from chronic pancreatitis (three patients) or repeat abdominal surgeries (one patient). Details of dosing scheme can be found in Supplementary Figure S4. Similar to our data in healthy individuals, S6 phosphorylation levels in monocytes and T cells drastically dropped within 1–5 h after intake of THC (**Figures 5A,B**). For two patients, we were able to collect longitudinal samples spanning the entire 50 days study period, including a pre-dose sample. Interestingly, in these two patients, we observed a very clear and constant increase in S6 phosphorylation in T cell compartment as well as in monocyte compartment over time during the study (**Figure 5C**). This increase in S6 phosphorylation remained, however, sensitive to short term treatment with medical cannabis (evident 1–5 h after intake of Namisol^®^). Thus, from our *in vitro* and *in vivo* data, the short term effects of THC include mTOR-S6 pathway downregulation, also in patients in chronic high THC regimen and thus in principle support use of medical cannabis for the treatment of chronic inflammatory diseases. However, our anecdotal evidence suggests that long term use of Namisol^®^ may increase levels of mTOR activity in T cells and monocytes, which indicates that the therapeutic dosing window for medical cannabis to combat inflammatory disease requires careful attention.

## Discussion

Medical marijuana is increasingly used to treat a variety of inflammatory diseases, but there is a paucity of mechanistic data that would support its use to combat inflammation *per se* (Gerich et al., 2015). In addition, its use in oncological disease is increasing, but again relatively little insight exists on how cannabis might influence the cancerous process (Chakravarti et al., 2014). Thus research characterizing these effects in humans is urgently needed. In this study, we employed kinome profiling to investigate the effect of medical marijuana on immune cell signaling in potentiated PBMCs. Using this unbiased approach (Li et al., 2012) we show that cannabis negatively modulates inflammatory signaling in immunocytes *in vivo*, and subsequent *in vitro* experimentation confirmed that these effects most likely derive from THC effects. We demonstrate that innate immune cell signaling is qualitatively, quantitatively and temporally different from adaptive cell signaling, but that phosphorylation of the immune signal S6 is decreased by THC in both monocytes and T cells. It is possible that total protein levels are affected by THC treatment, resulting in lower phosphorylation patterns. Unfortunately, material availability did not allow investigation of upstream mechanisms of S6 phosphorylation reduction. Signaling through mTOR-S6 in T cells has a well-known pro-inflammatory character (Pollizzi and Powell, 2015). However, in innate immune cells, mTOR-S6 signaling has also been suggested to reduce inflammatory responses (Weichhart and Säemann, 2009). Thus, the effect of inhibition of mTOR-S6 signaling may not be clear-cut. However, the overall effect appears to be a reduction of immunity, as perhaps best demonstrated by the use of mTOR inhibitors, such as the Rapamycin analogs Everolimus and Sirolimus, in the prevention of organ rejection after transplantation (Gong et al., 2015), although this may depend on the cell type contributing most to the immunological process at hand. Interestingly, HIV viral load was severely reduced in high-intensity cannabis users, supporting an immunomodulatory or antiviral effect of cannabinoids (Milloy et al., 2015). Altogether, our data appear to provide mechanistic support for the application of THC or cannabis preparations in the clinical management of diseases with an inflammatory component. However, it needs to be taken into account that whereas in a clinical setting inflammatory signals may already be present in patients newly receiving medicinal cannabis, in the current study cells were treated with THC prior to LPS stimulation, in order to obtain optimal potentiation of LPS signals.

To the best of our knowledge, this is the first report showing an acute downmodulation of mTOR-S6 signaling in immunocytes by cannabinoids. mTOR signaling has been studied before in response to cannabinoid receptor stimulation in other cell types, with conflicting results. Effects may depend on the location of receptor stimulation. CB1 and CB2 agonists increased mTOR-S6 signaling in oligodendrocytes (Gomez et al., 2011) and CB1 stimulation by THC resulted in transient mTOR activity in the brain (Puighermanal et al., 2009). In contrast, peripheral activity of CB1 receptor stimulation results in reduced mTOR activity, as was demonstrated by increased mTOR/S6K activation in gastric cells upon application of the CB1 antagonist rimonabant (Senin et al., 2013). This fits in with our finding of reduced mTOR signaling in peripheral blood cells upon cannabis application and thus the mechanistic insights obtained in the experimentation in volunteers and patients appear to have a more general relevance.

While we focused mainly on the anti-inflammatory molecular consequences of THC treatment of peripheral blood cells, mediated through p38 MAPK and mTOR-S6 signaling, several other interesting kinomic changes were observed upon cannabis intake in this study. For instance, a significant reduction of Wnt signaling was seen in potentiated PBMCs upon THC exposure. Wnt signaling is not only important for the regulation of embryogenesis, but also plays a role in cellular proliferation, migration and differentiation (van den Brink et al., 2004; Arias and Hayward, 2006). The Wnt signaling cascade has gained notoriety due to its deregulated nature in many cancers, where it is now regarded as target for treatment (Zhang and Hao, 2015). However, recent evidence suggests that Wnt may also regulate inflammation, in particularly by stimulating innate immunological responses, and aberrant activity and increased signaling is found in inflammatory disorders (George, 2008; Clevers and Nusse, 2012). Interestingly, while endogenous cannabinoids inhibit Wnt signaling in breast cancer cells (Laezza et al., 2012), this is the first time a Wnt inhibiting role for THC has been described in innate immune cells, demonstrating the strength of kinome profiling for identification of novel therapeutic targets.

Our data suggest that aside the aforementioned analgesic properties, the use of medical marijuana may provide additional benefits in patients with chronic inflammatory disorders. One of such diseases might be chronic pancreatitis (CP), a progressive fibroinflammatory disorder resulting in damage and scarring of pancreatic tissue, eventually leading to loss of function (Raimondi et al., 2010). Abdominal pain, the predominant symptom in CP, is in the early stage of the disease largely attributed to the interaction of inflammatory cells and neurons within the pancreas. The extensive pancreatic fibrosis found in CP is thought to be mediated by pancreatic stellate cells, activated by (necro)inflammation and/or alcohol (Witt et al., 2007). While the underlying immune pathology is not yet fully elucidated, it is clear that both the innate and adaptive immune response play a central role in the development of CP (Xue et al., 2014). In particular, increased numbers of mononuclear myeloid cells (i.e., monocytes, macrophages) as well as T cells are observed in CP and its stromal compartment (Jaskiewicz et al., 2003) and in animal models, suppression of T cells was shown to alleviate pancreatitis (Yamada et al., 2001). All things considered, a central role of inflammation in the pathogenesis of CP is implied. While we have previously shown that levels of the proinflammatory cytokine TNFα are increased in patients with CP, we did not observe an effect on Namisol^®^ intake on serum cytokine levels (Utomo et al., 2015). The current study suggests that Namisol^®^ may provide acute anti-inflammatory relief through down modulation of innate and adaptive inflammatory signaling. One potential mechanism of inflammatory signaling reduction by cannabinoids might be through the downregulation of the immune receptors recognizing the pathogen-associated molecular patterns (PAMPs), such as the LPS receptor Toll like receptor (TLR) 4 (Xu et al., 2007). Indeed, suppression of TLR4 mediated inflammatory signals by cannabinoids has been shown in many studies (reviewed in McCoy and Kathleen, 2016). However, anecdotal evidence in our study also suggests that mTOR-S6 signaling may increase in immunocytes upon prolonged exposure to THC. It is conceivable that repeated short term inhibition of signaling results in a compensatory upregulation of said pathway (see Supplementary Figure S5), arguing for caution in long-term use of such treatments. While not common, several case reports of THC-induced pancreatitis have been reported (Wargo et al., 2007; Mikolašević et al., 2013; Kayar et al., 2014), indicating that while short term THC use may be beneficial due to its anti-inflammatory properties, care should be taken with prolonged cannabis use.

## Ethics Statement

This study was part of two phase 2 trials using identical randomized, double-blind, placebo-controlled, parallel designs (clinicalTrials.gov ID: NCT01551511 and NCT01562483). The Medical Ethical Committee of the Radboud UMC approved the study and amendments for this additional study. The study was conducted according to the principles of the Declaration of Helsinki, and in accordance with the International Conference on Harmonization guidelines of Good Clinical Practice. All patients provided written informed consent.

## Author Contributions

WU, MdV, KP, and MC performed experiments. WU, MdV, KP, HB, MP, and GF analyzed data. WU, HB, MB, MP, HvG, and GF devised and supervised the study. WU, MP, and GF wrote the paper.

## Conflict of Interest Statement

The authors cooperated with Echo pharmaceuticals in a consortium conducting investigator-initiated phase 2 drug studies with Namisol^®^. The authors have no other relevant affiliations or financial involvement with any organization or entity with a financial interest in or financial conflict with the subject matter or materials discussed in the manuscript.
