# Supplementary material for: Modulation of Human Peripheral Blood Mononuclear Cell Signaling by Medicinal Cannabinoids
Source: Front Mol Neurosci. 2017 Jan 24;10:14. doi: 10.3389/fnmol.2017.00014 (PMC5258717; doi:10.3389/fnmol.2017.00014)
Supplement: Supplementary file 1 [file Table_1.DOCX]

**Supplementary Table 1. Patient lab scores at day 0, before Namisol® intake.**

|  | Mean | Range |
| --- | --- | --- |
| WBC | 9.9 | (8.4-12.3) |
| Thrombocytes | 244.3 | (204-303) |
| Red blood cells | 4.67 | (4.1-4.99) |
| Hemoglobin | 8.975 | (8.2-10.3) |
| Hematocrit | 0.41 | (0.38-0.46) |
| MCHC | 21.7 | (21.4-22.2) |
| MCV | 89 | (78-96) |
| MCH | 1.97 | (1.7-3.1) |
| Red cell distribution width | 13.9 | (12.8-15) |
| Albumin | 41.75 | (40-44) |
| Urate | 0.29 | (0.19-0.35) |
| Total bilirubin | 8.25 | (6-11) |
| Alkaline phosphatase | 83.25 | (34-118) |
| AST | 27.75 | (16-43) |
| ALT | 27.5 | (21-38) |
| LD | 180.75 | (159-221) |
| Gamma-GT | 65.5 | (13-160) |
| CK | 102.25 | (47-188) |
| Glucose | 4.8 | (4.5-5) |
| Sodium | 140.75 | (138-144) |
| Potassium | 3.8 | (3.5-3.9) |
| Calcium | 2.36 | (2.32-2.38) |
| Phosphate | 1.00 | (0.83-1.17) |
| Creatinine | 70.25 | (59-82) |

WBC: white blood cells, MCHC: mean corpuscular hemoglobin concentration; MCV: mean corpuscular volume; MCH: mean corpuscular hemoglobin; ASAT: Aspartate aminotransferase; ALAT: Alkaline aminotransferase ; gamma-GT: gamma-glutamyl transpeptidase; LD: lactate dehydrogenase ; CK: creatine kinase
