# Supplementary material for: Modulation of Human Peripheral Blood Mononuclear Cell Signaling by Medicinal Cannabinoids
Source: Front Mol Neurosci. 2017 Jan 24;10:14. doi: 10.3389/fnmol.2017.00014 (PMC5258717; doi:10.3389/fnmol.2017.00014)
Supplement: Supplementary file 3 [file Data_Sheet_1.docx]

**Supplementary Materials belonging to:**

Modulation of human peripheral blood mononuclear cell signaling by medicinal cannabinoids

Wesley K Utomo^1^, Marjan de Vries^2^, Henri Braat^1^, Marco J Bruno^1^, Kaushal Parikh^3^, Mònica Comalada^4^, Maikel P Peppelenbosch^1^, Harry van Goor^2^, Gwenny M Fuhler^1,*^

**Supplementary Figures and legends:**

Fig. S1. Primary results of the kinome profiling

Fig. S2. *In vitro* validation of direct THC effect on immune cells.

Fig. S3. Gating strategy of FACS analysis

Fig. S4. Dosing regimen of Namisol® in patients

Fig. S5. Speculated mTOR activity index upon THC treatment**.**

Data file Table S1. Patient lab scores at day 0, before Namisol® intake.

Data file Table S2: Kinomic changes of potentiated signal transduction in PBMCs before and after the intake of medical marijuana (separate file).

Supplementary Figures and legends

**Supplementary figure S1.** Primary results of the kinome profiling. Points represent the average of peptide phosphorylation from nine points (three technical replicas obtained from the blood of each of the three independent cannabis-naïve volunteers) of *ex* vivo LPS-challenged blood obtained before (X axis) and after (Y axis) ingestion of a medical cannabis preparation (Bedocran) by the volunteers.


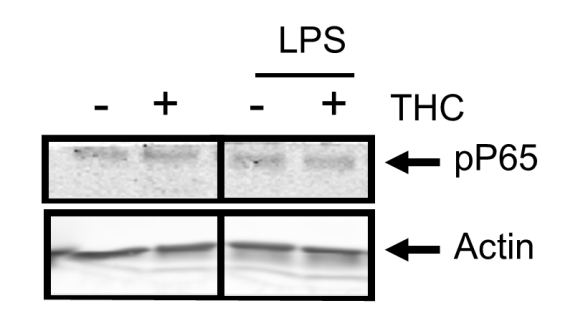


**Supplementary Figure S2.** PBMCs were isolated from healthy subjects (n=6) and after in vitro pretreatment with THC (2ng/mL) for 1h, cells were stimulated with 100 ng/mL lipopolysaccharide for 10 minutes. We were able to detect phosphorylated p65 by Western blot analysis in 3 experiments, none of which showed any modification of p65 phosphorylation upon THC treatment of cells. One example is shown. Samples were run on the same blot, and cropped, as indicated by the black line.


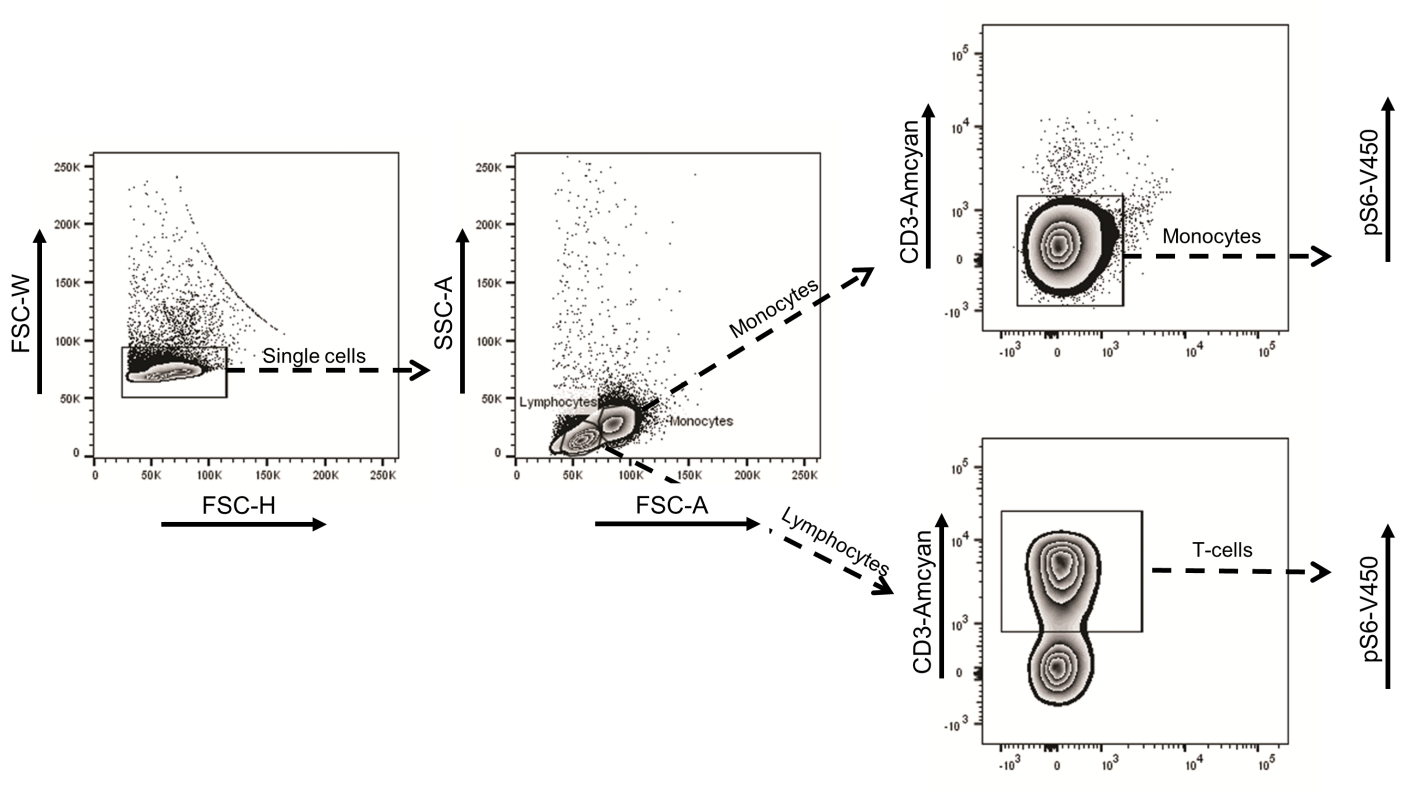


**Supplementary Figure S3**. Gating strategy intracellular pS6 analysis. Doublets were excluded from analysis by gating on FSC-W vs FCS-H. Single cells were subsequently gated for monocytes and T-cells based on their characteristic forward scatter and side-scatter patterns. Within the T-cell gate, CD3-Amcyan+ cells were gated. Within the monocyte gate, contaminating T-cells were excluded by CD3-amcyan exclusion. S6-V450 vs unused channel profiles are as shown in the remainder of the manuscript.


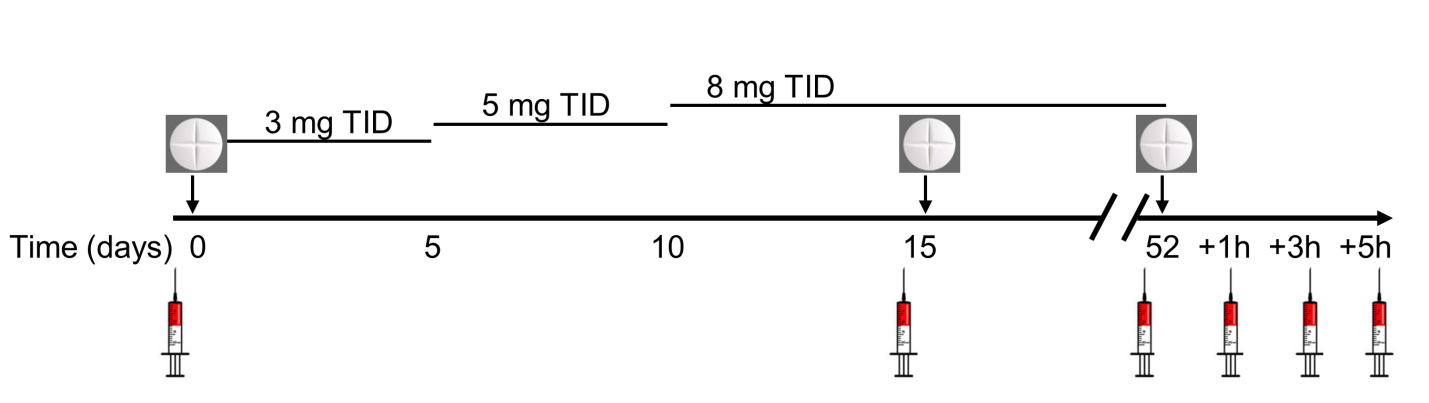


**Supplementary Figure S4**. Dosing regimen of Namisol® in patients. Blood was drawn predose at t=0, t=1 for two patients, and at day 52 predose and 1,3, and 5 h post dose for 4 patients.


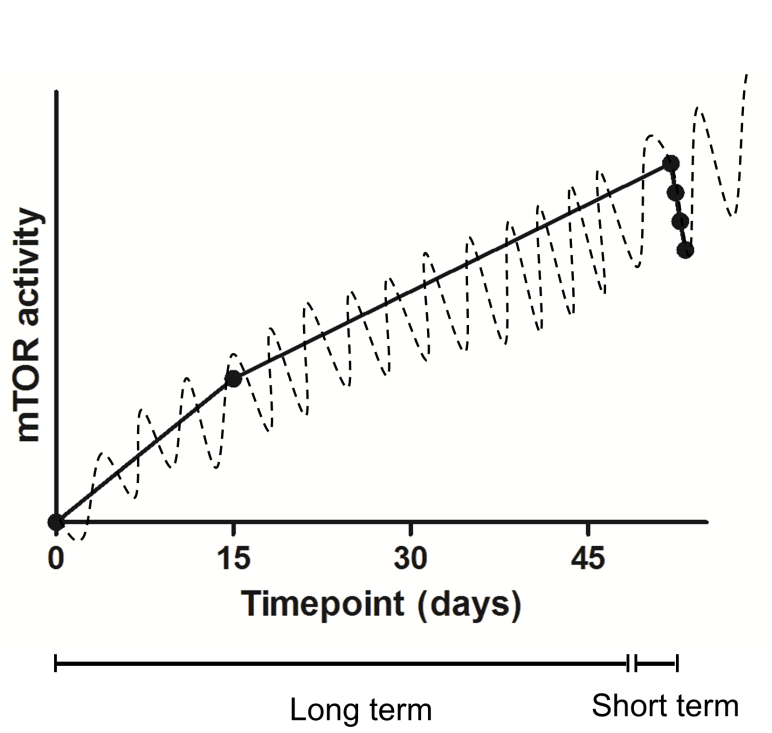


**Supplementary Figure S5**. Speculated mTOR activity index upon THC treatment**.** While mTOR pathway regulation is subject to downmodulation upon short term THC intake, prolonged THC treatment may increase basal mTOR-S6 levels in monocytes and T-cells.
